# Evaluation of the clinical performance of a risk scoring model for venous thromboembolism prophylaxis in cesarean section for a largely Chinese population

**DOI:** 10.1016/j.rpth.2026.103390

**Published:** 2026-02-17

**Authors:** Choi Wah Kong, William Wing Kee To

**Affiliations:** Department of Obstetrics and Gynaecology, United Christian Hospital, Kwun Tong, Hong Kong SAR

**Keywords:** cesarean section, China, pregnancy, risk score, venous thromboembolism

## Abstract

**Background:**

Recommendations for venous thromboembolism (VTE) prophylaxis after cesarean section (CS) from authoritative guidelines differed significantly and may not be applicable to Chinese population.

**Objectives:**

This study aimed to evaluate the performance and safety of an adapted risk score prophylaxis protocol in a largely Chinese cohort.

**Methods:**

The risk assessment and preventive care for VTE for CS protocol was implemented from May 2017 for all patients undergoing CS. Those with moderate risk (VTE score, 2) would be prescribed mechanical prophylaxis using pneumatic cuff, and those with high risk (VTE score, ≥3) would be prescribed pharmacologic prophylaxis with low-molecular-weight heparin in addition to the pneumatic cuff. In this retrospective cohort study, the outcome of patients with CS from the time of implementation of the protocol to December 2024 (7.5 years) was compared with that of a preprotocol group with no prophylactic measures from January 2014 to April 2017.

**Results:**

Among 4803 patients with CS in the protocol period, 633 (13.2%) women were prescribed mechanical prophylaxis and 156 (3.25%) patients required pharmacologic prophylaxis. Recoding the risk scores under the Royal College of Obstetricians and Gynaecologists or American College of Chest Physicians guidelines showed that 78.9% and 27.1% would require pharmacologic prophylaxis, respectively. There were 5 patients with VTE events in the protocol group compared with 11 among 3309 CS patients in the preprotocol group. The observed incidence of postpartum VTE significantly dropped from 0.33% to 0.1% after the implementation of the protocol (*P* = .043), while there was no increase in bleeding complications (0.96% in the preprotocol period vs 0.76% in the protocol period).

**Conclusion:**

Implementation of a standardized thromboprophylaxis protocol adapted for cesarean deliveries in our local obstetric population maintained pharmacologic thromboprophylaxis at a low rate and did not result in an increase in bleeding complications. A lower incidence of VTE was observed after implementation of the protocol than that in the preprotocol period.

## Essentials

• Pregnancy and CS increase the risk for VTE.
• A new risk assessment score is proposed for women undergoing CS.
• The new score maintained the use of pharmacologic thromboprophylaxis at a low rate.
• A lower incidence of VTE was observed after the implementation of the protocol.

## Graphical abstract

**Figure:**
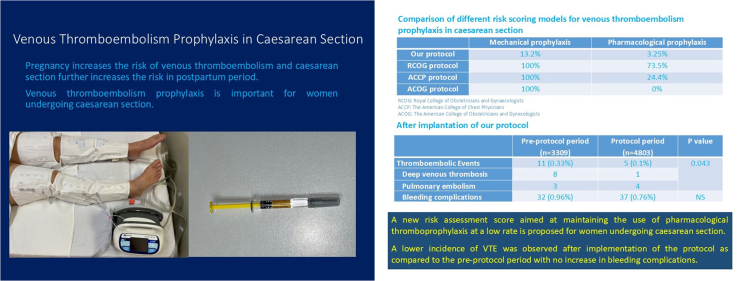

## 1 Introduction

Venous thromboembolism (VTE) remains a major cause of maternal mortality and morbidity [1]. The 2024 Mother and Babies; Reducing Risk through Audits and Confidential Enquiries across the United Kingdom (MBRRACE-UK) study showed that VTE accounted for 16% of maternal deaths during and up to 6 weeks after the end of pregnancy between 2020 and 2022 [2]. The relative risk for VTE among pregnant or postpartum women was shown to be 4 to 5 times higher than that for nonpregnant women, and the incidence was 5 times higher among postpartum women than pregnant women [3]. A significant fall in maternal mortality rate from pulmonary embolism (PE) from 1.56 to 0.70 per 100,000 maternities between 2003-2005 and 2006-2008 had been observed in the United Kingdom after the publication of the first Royal College of Obstetricians and Gynaecologists (RCOG) guidelines for VTE prophylaxis during pregnancy and the puerperium in 2004 [4], and this reduction was attributed to the successful use of VTE prophylaxis [5].

It remains controversial whether the incidence of VTE is lower [6,7] or similar in Asian women compared with western populations [8]. The types of thrombophilia that are more likely to contribute to VTE in Asian populations may also differ from that of western populations, with a higher incidence of congenital thrombophilia such as antithrombin, protein C, and protein S deficiencies in the Chinese population [9,10]. The risks of VTE have been demonstrated in a meta-analysis to be 4-fold higher after cesarean section (CS) than those after vaginal delivery [11], and up to 2.0% of patients suffering from DVT will have life-threatening PE [12]. Many authoritative protocols have been advocated for the prevention of VTE after CS [1,[13], [14], [15]], together with a variety of risk scoring systems [16]. However, there is considerable variation in these approaches to prophylaxis of VTE after CS, and the precise risk threshold at which pharmacologic prophylaxis of VTE is warranted remains controversial. RCOG recommends that all women who have had an intrapartum or urgent cesarean delivery meet the criteria for intermediate risk and should be considered for thromboprophylaxis for 10 days after delivery, while those having an elective CS should be considered for similar thromboprophylaxis if they have any additional risk factors [1]. The American College of Chest Physicians (ACCP) guidelines recommend prophylaxis with low-molecular-weight heparin (LMWH) when 1 major or 2 or more minor risk factors are present or when 1 minor risk factor is present in the setting of an emergency cesarean delivery [14]. The American College of Obstetricians and Gynecologists (ACOG) recommends universal pneumatic compression devices for all women undergoing CS, and if additional risk factors persist during the postpartum period, pharmacologic prophylaxis for up to 6 weeks should be considered [13]. The National Partnership for Maternal Safety recommends a consensus bundle, using universal pneumatic compression devices for all patients undergoing CS, and the addition of pharmacologic prophylaxis for women at high risk for VTE based on either the RCOG guidelines or the modified Caprini scoring system [16].

Given the significant discrepancies in the recommendations on using thromboprophylaxis from these guidelines and the possible variations in the incidence of VTE among different ethnic groups and among different obstetric populations, it has been recommended that each institution should develop a patient safety bundle with an institutional protocol for VTE prophylaxis for women undergoing cesarean delivery [17]. Our center has previously formulated an adapted guideline of VTE prophylaxis for CS modified from the RCOG guidelines. A pilot study involving 859 patients over 12 months using this protocol showed that we were able to reduce the rate of prescribing pharmacologic thromboprophylaxis to 3.3% compared with 75.6% if under the RCOG protocol, without any postpartum VTE events over the study period [18]. In this current study, we report our experience of using this protocol over 7.5 years. The much larger cohort would allow a more precise evaluation of the incidence of postpartum VTE events and the safety in terms of bleeding risks associated with pharmacologic thromboprophylaxis. In addition, a direct comparison of the incidence of VTE events before and after the implementation of this protocol was made to evaluate whether such a protocol could be effective in reducing VTE events in our CS patients.

## 2 Methods

The risk assessment and preventive care for VTE for CS protocol was implemented in the obstetrics unit of the United Christian Hospital from May 2017. The unit is a tertiary referral and training hospital in Hong Kong with on average ∼3000 deliveries per year during the study period. Before May 2017, there were no specific protocols for VTE prophylaxis for CS, and only general measures such as early postoperative mobilization were used without mechanical or pharmacologic thromboprophylaxis. A risk score model modified from the RCOG guidelines was implemented to assess the risk of VTE in women who undergo either elective or emergency cesarean delivery, while at the same time aiming to maintain the use of pharmacologic prophylaxis at a reasonably low rate. The model was based on demographic, obstetric, and medical risk factors of the patients (Table 1). Before undergoing CS, all patients would be scored in accordance with the assessment criteria. Those with moderate risk (VTE score, 2) would be prescribed mechanical prophylaxis using pneumatic cuff during CS, and the pneumatic cuff pumping will be continued postoperatively until the patients could fully mobilize. Those with major postpartum hemorrhage (PPH) of >1000 mL or who required blood transfusion during or immediately after CS would have their scores marked up by 1 point. Patients with high risk (VTE score, ≥3) after CS would be prescribed pharmacologic prophylaxis in the form of subcutaneous enoxaparin injection (40 mg in prefilled syringes) for 10 days in addition to the pneumatic cuff. The LMWH injection would be commenced at ∼12 hours after CS in those that were clinically stable with no ongoing primary PPH and no other medical contraindications. Any bleeding complications, such as significant wound hematomas requiring drainage or resuturing; secondary PPH (defined as delayed excessive bleeding after the first day of delivery); and any other major organ bleeding were specifically recorded [19]. The diagnosis of VTE is based on clinical features and confirmed by adequate radiological and ultrasound imaging as appropriate.

**Table 1:** Department guidelines of VTE prophylaxis for CS in the United Christian Hospital.

1) Patients who are on antenatal LMWH (such as previous DVT or high-risk thromboembolism) or those who have already planned for LMWH for 6 wk after delivery should have pneumatic cuff during CS and have LMWH after delivery as planned. Calculation of the VTE score will not be needed for these patients
2) Patient who will undergo elective or emergency CS should have the following risk factors checked (each risk factor score 1 point in the VTE score unless specified): • Age ≥ 40 y • BMI (prepregnancy or early pregnancy BMI), ≥25 but <30 kg/m^2^ • BMI ≥ 30 kg/m^2^ (scored as 2 in VTE score) • Parity ≥ 3 • Pre-eclampsia • Multiple pregnancy • Preterm gestation (<34 wk) • Prolonged labor (>24 h) • Stillbirth in this pregnancy • Medical comorbidities (eg, cancer, heart failure, active SLE, IBD or inflammatory polyarthropathy, nephrotic syndrome, type I DM with nephropathy, sickle cell disease, and current intravenous drug addiction) • Low-risk thrombophilia • Current smoker • Gross varicose veins • Current systemic infection • Immobility (eg, paraplegia) • A family history of VTE (first-degree relative)

Patients with a VTE score of ≥2 should have pneumatic cuff during CS. For patients with PPH of ≥1000 mL or have blood transfusion during CS, 1 point should be added in the VTE score. Patients with VTE score of ≥3 should be prescribed at least 10 days postnatal prophylactic LMWH. Regimen—maternal booking or early pregnancy body weight of <100 kg: enoxaparin subcutaneous 40 mg daily; maternal booking or early pregnancy body weight of ≥100 kg: enoxaparin subcutaneous 60 mg daily.

BMI, body mass index; CS, cesarean section; DM, diabetes mellitus; IBD, inflammatory bowel disease; LMWH, low-molecular-weight heparin; PPH, postpartum hemorrhage; SLE, systemic lupus erythematous; VTE, venous thromboembolism.

Data on risk scores, the need for thromboprophylaxis, perinatal outcome, and any postpartum VTE events were collected retrospectively for all CS patients from the time of commencement of this protocol to December 2024. Demographic data, gestational age at delivery, and postpartum complications were retrieved from the Hospital Authority electronic medical record system. Data on all VTE events diagnosed clinically and documented in our clinical management system from the time of delivery up to 6 weeks postpartum wound be included. In addition, an extended search on the Hospital Authority Clinical Data Analysis and Reporting System was made for all recruited patients for unplanned readmissions either to our hospital or to other public institutions, up to 6 weeks after their CS to pick up any delayed VTE events that occurred only after the index admission. The rate of mechanical and pharmacologic prophylaxis for CS in our risk score protocol was compared with the RCOG and the ACOG and ACCP guidelines as applied to our patients.

The perinatal outcome of all patients with cesarean deliveries from January 2014 to April 2017 were collected to serve as a historical preprotocol control group. The detailed perinatal risk factors for thromboembolism were reviewed retrospectively using the Hospital Authority Obstetrics Clinical Information System, as well as manually as needed. Risk scores were retrospectively calculated based on our protocol, as well as with other protocols as in study patients, and the hypothetical rate of using mechanical or pharmacologic thromboprophylaxis was calculated. Apart from VTE complications that were documented in the records, an extended search was similarly performed on all recruited cases to pick up any admissions up to 6 weeks after their index admission.

The study was approved by the research ethics committee of the hospital. Being a retrospective cohort study, individual patient consent was waived. Descriptive statistics were performed including means with SDs and proportions, and differences between incidences were compared using chi-squared and Fisher exact tests as appropriate. A *P* value of < .05 was considered statistically significant. The Statistical Package for Social Sciences for Windows package, version 28, was used for data entry and analysis.

## 3 Results

There was a total of 20,027 deliveries from the start of our protocol in May 2017 to December 2024 (92 months), of which 4817 deliveries (24%) underwent CS. Within the preprotocol control period from 2014 to April 2017 (40 months), there were a total of 14,212 deliveries, of which 3318 deliveries (23.3%) were delivered by CS. Among these patients, 14 in the protocol group and 9 in the preprotocol group were already on LMWH in the antenatal period for specific medical indications, including 14 with acquired antiphospholipid syndrome, 6 with a history of previous pregnancy-related DVT, and 3 with hereditary thrombophilia. While LMWH was continued after CS in all 23 cases, these were excluded from the subsequent comparison analysis. The protocol cohort therefore consisted of 4803 patients, while the preprotocol group consisted of 3309 patients.

### 3.1 Comparison of clinical characteristics

The demographic, obstetric, and medical characteristics of the preprotocol and protocol groups were compared (Table 2). There was a similar proportion of women of non-Chinese ethnicity in both periods of ∼5%. Remarkably, there were higher proportions of patients with overweight (BMI >25 but <30 kg/m^2^) and obesity (BMI > 30 kg/m^2^) in the protocol period (13% and 4.97%, respectively) than those in the preprotocol group (10.9% and 3.72%, respectively; *P* < .001), indicating a trend of increasing obesity in recent years. While the proportion of women conceived by assisted reproductive technology remained constant, the number of multiple pregnancies were significantly lower in the protocol period (4.7% vs 6%) and could be related to the wider current practice of single embryo transfer. In addition, as patients with active COVID infection at the time of delivery would be labeled as having current systemic infection, there was a higher incidence of current systemic infection in the protocol period (0.33% vs 0.06%) than that of the preprotocol years before the outbreak of COVID-19. There was a significantly higher incidence of primary PPH in the protocol group (6.05% vs 4.01%), reflecting the general trend of increasing PPH rates in Hong Kong [20,21]. Other medical risks for thromboembolism were similar between the 2 groups (Table 2).

**Table 2:** Demographic, obstetric, and medical characteristics of the patients managed under the risk assessment and preventive care model.

Factor	Preprotocol period (*n* = 3309)	Protocol period (*n* = 4803)	*P*
Demographic factors			
Ethnicity			
Chinese	3110 (94)	4575 (95.26)	1.17
Non-Chinese	175 (5.28)	228 (4.74)	
Filipino/South Asian	143 (4.32)	176 (3.67)	
White	32 (0.96)	52 (1.08)	
Maternal age ≥ 40 y	333 (10.1)	537 (11.2)	.11
Parity ≥ 3	1 (0.03)	8 (0.16)	.09
Assisted reproductive technology	215 (6.5)	330 (6.87)	.24
Prepregnancy BMI, ≥25 but <30 kg/m^2^	361 (10.9)	624 (13)	.005^a^
Prepregnancy BMI, ≥30 kg/m^2^	123 (3.72)	239 (4.97)	.007^a^
Preterm delivery < 34 wk	335 (10.1)	480 (9.99)	.85
Obstetric factors			
Pre-eclampsia	99 (2.99)	161 (3.35)	.40
Multiple pregnancy	199 (6)	226 (4.7)	.01^a^
Prolonged labor >24 h	36 (1.08)	38 (0.79)	.19
Stillbirth	3 (0.09)	2 (0.04)	.40
Elective CS	1408 (42.5)	1987 (41.4)	
Emergency CS	1901 (57.5)	2816 (58.6)	.29
PPH ≥ 1000 mL or required blood transfusion	133 (4.01)	291 (6.05)	.001^a^
Medical factors			
Medical comorbidities—total	34 (1)	55 (1.14)	.61
Systemic lupus erythematous	28	43	
Cancer	3	5	
Crohn disease	1	3	
Heart disease	2	4	
Low-risk thrombophilia	5 (0.15)	11 (0.23)	.62
Current smoker	29 (0.87)	30 (0.62)	.15
Gross varicose veins	2 (0.06)	3 (0.06)	1.0
Current systemic infection	2 (0.06)	16 (0.33)	.014^a^
Immobility (eg, paraplegia)	0	0	—
Family history of VTE (first-degree relative)	6 (0.18)	4 (0.08)	.33

Values are *n* (%).

BMI, body mass index; LMWH, low-molecular-weight heparin; PPH, postpartum hemorrhage; VTE, venous thromboembolism.

a Statistically significant.

### 3.2 Risk scoring

In the protocol group, 4064 (84.6%) patients with a VTE score of ≤1 were only advised early mobilization after CS. Of 583 women with a VTE final score of 2, 477 patients had this score before CS and so were prescribed mechanical prophylaxis with pneumatic calf pumping during and after CS, while 106 patients had a risk score of only 1 before CS and only had a score of 2 after delivery due to major PPH and/or required blood transfusion. As the latter group did not have mechanical prophylaxis during operation, they were excluded as having mechanical prophylaxis. Of 156 (3.25%) patients with VTE score of ≥3 before or after CS, all were prescribed mechanical prophylaxis during and after CS, 153 were prescribed pharmacologic prophylaxis with LMWH after CS, while 3 were given therapeutic doses of LMWH as they were already diagnosed to have VTE in the early postoperative period. When these patients were recoded according to the RCOG protocol, 73.5% and 78.9% of them in the preprotocol and protocol groups would theoretically be prescribed pharmacologic prophylaxis. On the contrary, when they were recoded according to the ACOG protocol, as those with a history of VTE or thrombophilia who were already on LMWH before CS were excluded, none of them would be prescribed pharmacologic prophylaxis. Using ACCP guidelines, 24.4% in the preprotocol group and 27.1% in the protocol group would require pharmacologic thromboprophylaxis, which were still considerably higher compared with our current protocol.

### 3.3 VTE events

A total of 11 postpartum thromboembolic events were documented in the preprotocol group, among which there were 3 cases of PE and 8 cases of deep venous thrombosis. There were 5 cases documented in the protocol period, 4 with PE and 1 with deep venous thrombosis. The incidence of VTE events after the implementation of the current protocol (0.1%) was significantly lower than that of the preprotocol period without prophylaxis (0.33%; *P* = .043) (Table 3), with an overall incidence of VTE of 1.9 per 1000 patients with CS in the entire cohort. No patients who received LMWH prophylaxis after CS had serious hemorrhage or complications that could be directly related to the LMWH used. The wound complication rates and secondary PPH rates were comparable between the 2 groups. No case of heparin induced thrombocytopenia was documented in the prophylaxis group (Table 3). All patients with VTE recovered fully, and there was no maternal mortality related to VTE in either the protocol or preprotocol period.

**Table 3:** Scoring and the rate of mechanical and pharmacologic prophylaxis used under different guidelines.

Guideline	Preprotocol period (*n* = 3309)	Protocol period (*n* = 4803)
Current protocol		
Risk score		
0	2026 (61.2)	2775 (57.8)
1	857 (25.9)	1289 (26.8)
2	322 (9.73)	583 (12.1)
3	88 (2.66)	132 (2.75)
≥4	16 (0.12)	24 (0.5)
Mechanical prophylaxis	—	633 (13.2)
Pharmacologic prophylaxis	—	153 (3.18)
Hypothetical prophylaxis rates under RCOG protocol		
Mechanical prophylaxis	3309 (100)	4803 (100)
Pharmacologic prophylaxis	2432 (73.5)	3789 (78.9)
Hypothetical prophylaxis rates under ACCP protocol		
Mechanical prophylaxis	3309 (100)	4803 (100)
Pharmacologic prophylaxis	809 (24.4)	1301 (27.1)
Hypothetical prophylaxis rates under ACOG protocol		
Mechanical prophylaxis	3309 (100)	4803 (100)
Pharmacologic prophylaxis	0	0
Outcome		
Thromboembolic events	11 (0.33)^a^	5 (0.1)^a^
Deep venous thrombosis	8 (0.24)	1 (0.02)
Pulmonary embolism	3 (0.09)	4 (0.08)
Bleeding complications	32 (0.96)	37 (0.76)
Wound infection/hematoma requiring resuturing	26 (0.78)	33 (0.68)
Secondary postpartum hemorrhage	6 (0.18)	4 (0.08)
Major organ bleeding	0	0

Values are *n* (%).

ACCP, American College of Chest Physicians; ACOG, American College of Obstetricians and Gynecologists; RCOG, Royal College of Obstetricians and Gynaecologists.

a *P* = .043.

The clinical details of all cases with documented thromboembolic postpartum events were tabulated in Table 4. Reviewing the retrospective risk scores in the VTE cases in the preprotocol group, 8 of them would have received pneumatic cuffs and 5 would have been prescribed LMWH within the first day if the current protocol had been adapted. For the 5 VTE cases in the protocol group, 4 had pneumatic cuffs during CS, and 3 should be prescribed LMWH after delivery. However, the commencement of LMWH was delayed in all 3 cases, as there was concern about PPH or actual severe PPH in these cases. It remains debatable whether earlier commencement of LMWH in these patients could have prevented the VTE events. Furthermore, in case 5, there was suspicion that the patient had already developed DVT at the time pneumatic cuffs were applied, so that prophylaxis would no longer be appropriate.

**Table 4:** Cases with venous thromboembolic events.

Case	Age (y)	Risk score	Clinical presentation
Preprotocol period			
Case 1	29	Score =2 BMI, 30 kg/m^2^	Parity 1. Elective repeat LSCS for previous CS at term; intraoperative desaturation. ECG showed typical S1Q3T3. Urgent CTPA showed filling defects in lobar and segmental pulmonary arteries. USG showed no DVT.
Case 2	31	Score = 3 BMI, 27 kg/m^2^/preterm/PPH	LSCS at 29+ wk for preterm prelabor rupture of membranes; PPH with blood loss of 1 L; discharged on day 5 but readmitted on day 7 for hemoptysis. CTPA findings were compatible with pulmonary embolism
Case 3	43	Score = 2 Age/PPH	Parity 2. Elective LSCS for 2 previous CS; PPH with blood loss of 1.1 L and uterine atony, controlled by oxytoxics; presented with tachycardia and shortness of breath on day 2. CTPA showed pulmonary emboli in left interlobar arteries.
Case 4	26	Score = 0	Parity 2. Repeat LSCS for 2 previous CS; unilateral lower–limb swelling 7 d postdelivery. Doppler USG confirmed left-calf DVT, treated with LMWH for 6 wk.
Case 5	40	Score = 2 Age/medical comorbidity	Parity 1. A history of breast carcinoma with right mastectomy and left cephalic vein thrombosis related to central line insertion for chemotherapy; elective LSCS for previous CS at 39 wk; left femoral DVT confirmed by Doppler USG, presenting on day 5 postdelivery
Case 6	45	Score = 3 Age/BMI, 31 kg/m^2^	Parity 1. Elective LSCS for previous CS; bilateral lower–limb swelling on day 5 postoperatively. USG showed left popliteal DVT.
Case 7	42	Score = 3 Age/BMI, 30 kg/m^2^	Parity 0. IVF pregnancy and elective LSCS for breech; left lower–limb swelling 4 wk after delivery. USG confirmed left-calf DVT.
Case 8	46	Score = 3 Age/twin/PET	Parity 0. IVF pregnancy, LSCS for PET, twin pregnancy; desaturation with shortness of breath on day 3 postdelivery. CTPA showed filling defects in right pulmonary branches. Echocardiogram showed features of right heart strain, treated as pulmonary embolism with LMWH. USG showed no lower-limb DVT
Case 9	27	Score = 1 Medical comorbidity	Parity 0. A history of epilepsy treated with regular antiepileptic drugs; elective LSCS for malpresentation; readmitted on day 8 with left lower–limb swelling. USG showed left common femoral vein thrombosis, subsequently diagnosed with protein S deficiency.
Case 10	38	Score = 3 PET/BMI, 28 kg/m^2^/PPH	Parity 0. IVF pregnancy with elective LSCS for previous myomectomy. Dense adhesions were encountered with PPH due to uterine atony of 1.2 L; left lower–limb swelling 4 wk after delivery. USG confirmed left lower–limb DVT.
Case 11	38	Score = 1 BMI, 27 kg/m^2^	Parity 1. Elective LSCS for previous CS; lower-limb swelling on day 3 postoperatively. USG showed left-calf and popliteal DVT.
Protocol period			
Case 1	29	Score =2 PET/BMI, 27 kg/m^2^	Parity 0. Induction of labor at 40 wk for PET; emergency LSCS for failed induction; clinical sepsis with swinging fever immediately postoperatively. Day 2 bilateral lower–limb Doppler showed no DVT. CTPA showed filling defects within segmental branches of right pulmonary artery, treated with LMWH from day 3.
Case 2	39	Score = 4 PET/BMI, 29 kg/m^2^/stillbirth/preterm	Parity 0. Early-onset PET and fetal growth restriction; multiple large fibroids; stillbirth at 31 wk. Emergency LSCS done for failed induction and oblique breech presentation. Persistent tachycardia observed postoperatively. CTPA on day 3 showed central filling defects in segmental pulmonary arteries, treated with LMWH.
Case 3	39	Score = 4 BMI, 31 kg/m^2^/preterm/PPH	Parity 1. Multiple large uterine fibroids occupying lower segment; emergency classical CS for antepartum hemorrhage and transverse lie at 33 wk; PPH up to 3 L controlled with intrauterine balloon tamponade; desaturation on day 2. CTPA confirmed main pulmonary trunk embolism. No DVT oserved on USG Doppler. Emergency pulmonary thrombectomy was done and then started on LMWH.
Case 4	40	Score = 3 Age/PPH/BMI, 29 kg/m^2^	Parity 1. Elective repeat LSCS at 39 wk for previous CS; PPH with blood loss of 1 L controlled with oxytoxics; intraoperative desaturation treated as pulmonary embolism with LMWH. Urgent CTPA showed filling defects at interlobar right pulmonary artery.
Case 5	40	Score = 1 Age/BMI, 28 kg/m^2^	Parity 1. Repeat LSCS at 38 wk for previous CS in labor; prescribed pneumatic cuffs during operation but pumping withheld when unequal lower-limb swelling noticed. Postoperative USG confirmed right popliteal DVT.

BMI, body mass index; CS, cesarean section; CTPA, computed tomography pulmonary angiogram; DOAC, direct oral anticoagulant; DVT, deep venous thrombosis; ECG, electrocardiogram; IVF, *in vitro* fertilization; LSCS, lower segment CS; PET, pre-eclampsia; PPH, postpartum hemorrhage; USG, ultrasound.

According to the risk scoring in the protocol group, the incidence of VTE in those with score of <2, 2, and ≥3 were 0.02% (1/4064), 0.17% (1/583), and 1.9% (3/156), respectively. If we treat the current risk scoring as a screening test in the preprotocol group where there was no intervention, taking those with high risk who were prescribed pharmacologic prophylaxis as being screened positive, and those with postpartum VTE events as true positives, the sensitivity of the current scoring would be 45.5% (95% CI, 16.7%-76.6%); specificity, 97.1% (95% CI, 96.5%-97.7%); positive predictive value, 5% (95% CI, 2.6%-9.4%); and negative predictive value, 99.8% (95% CI, 99.7%-99.9%), with an overall accuracy of 97% (95% CI, 96.3%-97.5%). When the entire cohort over the 11 years was included, and disregarding the effects of intervention, the sensitivity would be 56.3% (95% CI, 29.9%-80.3%); specificity, 96.9% (95% CI, 96.5%-97.3%); positive predictive value, 3.5% (95% CI, 2.3%-5.4%); and negative predictive value, 99.9% (95% CI, 99.8%-99.95%), with an overall accuracy of 96.8% (95% CI, 96.4%-97.2%).

## 4 Discussion

Our overall incidence of VTE after CS of ∼0.2% (11+5/8112) was on par with the incidence reported from other studies based on western obstetric populations of ∼0.2% [22,23]. Our comparative scoring confirmed that adopting either the RCOG or ACCP criteria would entail a much higher rate of pharmacologic prophylaxis. We were able to demonstrate an apparent reduction in VTE episodes after the implementation of our protocol from 0.33% to 0.1%. Within the entire 10-year study period, excluding those that were already prescribed pharmacologic prophylaxis in the antenatal period, we were able to identify only 3 patients with VTE after vaginal delivery, confirming a much higher VTE risk after CS compared with vaginal births.

Compared with the RCOG guidelines, our current protocol has adopted a higher maternal age cutoff of 40 years instead of 35 years, a lower BMI cutoff between 25 and 30 kg/m^2^ as overweight to gear to Asian body habitus, and a preterm delivery cutoff of ≤34 weeks instead of 37 weeks. More importantly, instead of initiating pharmacologic prophylaxis for patients with 2 minor risk factors or more, our protocol raised the scoring threshold to 3 or above before prescribing LMWH. Compared with only early mobilization after CS in the preprotocol era, we were able to demonstrate a significant reduction of VTE events to ∼1 per 1000 women after adoption of the protocol despite a low rate of LMWH prophylaxis of only 3.25%.

Many studies have highlighted the wide discrepancies in the recommended prophylaxis between the major guidelines. These different guidelines have brought about as much controversy as they have consensus, as most of the recommendations are based not on incontrovertible evidence but largely on low level evidence from expert opinion, observational studies, and extrapolation from nonobstetric populations [24]. Similar to our findings, in a small cohort, it was found that the RCOG guidelines categorized 40.1% of all deliveries and 88.8% of CS as requiring LMWH thromboprophylaxis, while the ACOG and ACCP guidelines categorized 35.0% and 40.0%, respectively [22]. In another study including women delivered both vaginally or by CS, by applying RCOG guidelines, 53.6% of women were qualified for postpartum LMWH, compared with 40.2% of women using the Society of Obstetric Medicine of Australia and New Zealand guidelines, 37.3% using Society of Obstetricians and Gynaecologists of Canada guidelines, and 8.3% using ACCP guidelines [25]. In a cohort with only cesarean deliveries, 85.0% of women would need LMWH prophylaxis under RCOG recommendations compared with 1.0% under ACOG guidelines and 34.8% under ACCP guidelines [26]. The risk thresholds to recommend the use of LMWH in many of these guidelines appear very low and may probably imply an unjustifiably high number needed to treat to prevent VTE events. However, practical calculations of such numbers needed to treat or to harm were universally lacking [27], with almost no data from randomized controlled trials or meta-analyses to support recommendations [24]. There is a genuine concern that many current guidelines might lead to overmedicalization of pregnancy [28] with potential deleterious effects. Based on our data, the estimated risk reduction in VTE from preprotocol to the protocol period would be ∼0.2%, so that the number needed to be screened would be ∼500 per VTE event. Assuming the average rate for pharmacologic thromboprophylaxis using this risk scoring was in the range of 3% to 5%, the number needed to treat with LMWH to prevent 1 VTE would be in the reasonable range of 15 to 25.

Studies on decision analysis modeling found that there is substantial decision uncertainty regarding the selection of high-risk women for postpartum prophylaxis, mainly around the effectiveness of thromboprophylaxis for preventing VTE [29]. Using a hybrid decision tree model for North American women, it was estimated that adoption of the ACOG guidelines with a low LMWH use of 87 to 115 doses per 1000 cesarean deliveries would avert ∼5% of VTE cases, the ACCP guidelines would use 570 doses per 1000 deliveries and reduce up to 21.2% of VTE cases, while the RCOG guidelines would use over 7233 does per 1000 deliveries to avert up to 37.4% of VTE cases, and a universal regime of LMWH for 6 weeks postpartum would require an overwhelming 38,648 doses of LMWH per 1000 patients and avert 57.6% of all VTE [30]. A study adopting the ACOG District II Safe Motherhood Initiative’s VTE Bundle to guide heparin-based thromboprophylaxis showed that while more patients received LMWH prophylaxis postprotocol (15.6% vs 1.2%), there was no difference in VTE incidence (0.1% overall) between the preprotocol and postprotocol groups among a total of 24,000 deliveries. However, patients in the postprotocol group experienced significantly more wound hematomas, unplanned surgical procedures, and blood transfusions [31]. Furthermore, a meta-analysis including 6 randomized control trials and 1 prospective cohort showed that LMWH was associated with no obvious decrease in the risk of VTE but was associated with a relative risk of 8.47 for bleeding or hematomas in comparison with that in the negative control [32]. The 2014 Cochrane review also concluded that there is insufficient evidence on which to base recommendations for thromboprophylaxis during the early postnatal/postcesarean period, due to the limited number of trials and that these were not of high methodological quality [33].

The potential association of LMWH use with increased rates of postpartum complications remains a major clinical concern. Among postpartum women who received early therapeutic anticoagulation, the incidence of a composite of major hemorrhagic complications or major wound complications was reported as 8.4% for cesarean deliveries and 6.0% for vaginal deliveries. Complications were associated with earlier resumption of therapeutic anticoagulation, particularly before 9.25 hours for vaginal deliveries and before 15.1 hours for CS deliveries [34]. In contrast, a study on cases receiving enoxaparin prophylaxis within 24 hours of CS showed that, irrespective of the presence of an epidural catheter, no patient developed a spinal epidural hematoma after early administration of enoxaparin. In addition, the overall incidence of hemorrhagic complications did not increase [35]. We did not experience any of the major complications mentioned from the use of LMWH thromboprophylaxis in our current cohort. However, as discussed, overconservative practices and undue delays in commencing LMWH after CS could contrarily lead to prophylaxis failure. Given the conflicting results, the optimal time for starting LMWH thromboprophylaxis after a CS would probably need to be based on large, randomized trials.

Previous studies have shown that intermittent pneumatic compression can help to reduce the incidence of deep venous thrombosis, but the cost of intermittent pneumatic compression was only a small fraction of pharmacologic thromboprophylaxis [36]. Many guidelines, including the RCOG and ACOG, recommend universal use of pneumatic cuffs in all cesarean deliveries [1,13]. The use of mechanical prophylaxis with sequential compression devices is an inexpensive, safe intervention and should be used in all women undergoing cesarean delivery until the woman is fully ambulatory. New models of such devices are also more convenient to use and maintain. Therefore, with the availability of more such devices in our wards, instead of prescribing pneumatic cuffs only for those with a VTE score of ≤2, we are also moving toward universal usage of mechanical prophylaxis as an enhancement to our protocol.

There were limitations and strengths to our current study. While we searched for all postpartum VTE events within our system, we recognized that there could still be a possibility that we could have underestimated the VTE incidence. We could also have underdiagnosed asymptomatic VTE cases, but we do not believe that there is a need to routinely screen asymptomatic postpartum patients without clinical morbidity. On the contrary, despite the increasing incidence of maternal obesity, primary PPH, and other risk factors for VTE in our protocol cohort, we were still able to demonstrate an apparent reduction in VTE rates after implementation of our protocol. Nevertheless, due to the low incidence of VTE events, it was not surprising that significant reductions in its incidence could be difficult to demonstrate even in large cohorts [31,32] and that the possibly of chance findings posed from small number variations need to be borne in mind. Although we observed a significantly lower incidence of VTE events after implementation of the protocol, this should not be implied as causative. Previous authoritative meta-analyses have concluded that there is insufficient evidence on which to base recommendations for thromboprophylaxis in pregnancy and the early postpartum period [33]. There is a need for large, randomized controlled studies on the efficacy of pharmacologic thromboprophylaxis in reducing VTE events in CS patients, and trials on specific high-risk women have been proposed [29].

## 5 Conclusion

The decision to prescribe mechanical and pharmacologic prophylaxis in women undergoing cesarean deliveries should be based on a standardized scoring system geared to the incidence of postpartum VTE for the specific obstetric population. Our experience in implementing such a protocol showed that bleeding complications were not increased while pharmacologic thromboprophylaxis could be maintained at a low rate. A lower incidence of VTE was observed after implementation of the protocol than that in the preprotocol period.
